# Celecoxib, a Non-Steroidal Anti-Inflammatory Drug, Exerts a Toxic Effect on Human Melanoma Cells Grown as 2D and 3D Cell Cultures

**DOI:** 10.3390/life13041067

**Published:** 2023-04-21

**Authors:** Alessandro Venuta, Rosarita Nasso, Armando Gisonna, Roberta Iuliano, Sara Montesarchio, Vittoria Acampora, Leandra Sepe, Angelica Avagliano, Rosaria Arcone, Alessandro Arcucci, Maria Rosaria Ruocco

**Affiliations:** 1 Department of Public Health, University of Naples Federico II, 80131 Naples, Italy; alessandro.venuta@unina.it (A.V.); vittoria.acampora@unina.it (V.A.); angelica.avagliano@gmail.com (A.A.); 2 Department of Movement Sciences and Wellness, University of Naples “Parthenope”, 80133 Naples, Italy; rosaritanasso@gmail.com (R.N.); rosaria.arcone@uniparthenope.it (R.A.); 3 Department of Molecular Medicine and Medical Biotechnology, University of Naples Federico II, 80131 Naples, Italy; ar.gisonna@studenti.unina.it (A.G.); roberta.iuliano2@studenti.unina.it (R.I.); sar.montesarchio@studenti.unina.it (S.M.); leandra.sepe@unina.it (L.S.)

**Keywords:** non-steroidal anti-inflammatory drugs, celecoxib, melanoma, two-dimensional (2D) cell cultures, three-dimensional (3D) cell cultures

## Abstract

Cutaneous melanoma (CM) remains one of the leading causes of tumor mortality due to its high metastatic spread. CM growth is influenced by inflammation regulated by prostaglandins (PGs) whose synthesis is catalyzed by cyclooxygenases (COXs). COX inhibitors, including non-steroidal anti-inflammatory drugs (NSAIDs), can inhibit tumor development and growth. In particular, in vitro experiments have shown that celecoxib, a NSAID, inhibits the growth of some tumor cell lines. However, two-dimensional (2D) cell cultures, used in traditional in vitro anticancer assays, often show poor efficacy due to a lack of an in vivo like cellular environment. Three-dimensional (3D) cell cultures, such as spheroids, are better models because they can mimic the common features displayed by human solid tumors. Hence, in this study, we evaluated the anti-neoplastic potential of celecoxib, in both 2D and 3D cell cultures of A2058 and SAN melanoma cell lines. In particular, celecoxib reduced the cell viability and migratory capability and triggered the apoptosis of melanoma cells grown as 2D cultures. When celecoxib was tested on 3D melanoma cell cultures, the drug exerted an inhibitory effect on cell outgrowth from spheroids and reduced the invasiveness of melanoma cell spheroids into the hydrogel matrix. This work suggests that celecoxib could represent a new potential therapeutic approach in melanoma therapy.

## 1. Introduction

Among solid tumors, cutaneous melanoma (CM) is one of the most aggressive forms and represents one of the leading causes of cancer-related mortality because of its high metastatic spread [1]. It is known that the development and progression of melanoma is strictly linked to specific genomic alterations, generally occurring in cells of somatic tissues and involving both oncogenes and tumor suppressor genes [2,3].

Exogenous factors play a pivotal role in the development of melanoma. Indeed, exposure to ultraviolet radiation (UV) can lead to genomic damage that contributes to uncontrolled melanocytic proliferation [4,5]. In particular, mutations in the B-Raf Proto-Oncogene, Serine/Threonine Kinase (BRAF) gene or Neuroblastoma RAS Viral Oncogene Homolog (NRAS) gene have been detected in most CM cases, with a frequency of 50–70% and 15–30%, respectively [1].

In its early stage, melanoma is treated promptly by a surgical excision, which represents the most common way to eradicate melanomas [6]. However, when melanoma is diagnosed in an advanced stage, the surgical treatment must be associated with systemic standard therapies [7]. In particular, molecular targeted therapy with BRAF and/or MEK inhibitors (BRAF/MEKi) represents one of the most promising therapeutic strategies against CM-carrying mutations in B-RAF or N-RAS genes. However, the overall survival (OV) is limited in most patients because of the occurrence of therapy resistance due to high melanoma plasticity that induces the activation of alternative survival pathways [8,9,10,11]. In particular, drug treatments can induce genomic and non-genomic modifications that lead to MAPK signaling reactivation and/or AKT pathway sustainment [12]. Therefore, there is an urgent need to find other molecules that have an effect against melanoma, even when used in association with current anti-melanoma drugs.

Besides genomic instability, inflammation represents a pivotal factor in tumor development and progression. In fact, inflammatory factors such as cytokines, chemokines, and prostaglandins (PGs) are secreted by the tumor cells and generate an optimal environment for cancer development and progression [13,14]. In particular, skin is exposed to several environmental factors that provoke a chronic inflammation status often associated with the onset of tumorigenesis [15]. PGs are the main molecules promoting the inflammatory processes, and their production is catalyzed by cyclooxygenases (COXs), starting from arachidonic acid. There are two forms of COXs: COX-1 is constitutively expressed in most cells, whereas COX-2, the inducible form, is rapidly expressed by inflammatory stimuli, mitogens, growth factors, and cytokines [16]. It is known that the increased expression of COX-2 in many cancer cells is associated with increments of proliferation, angiogenesis, and metastasis that are mediated by prostacyclins and prostaglandins [17,18,19]. In particular, COX-2 can be considered as a negative prognostic marker for many tumors, including melanoma [20,21,22]. Non-steroidal anti-inflammatory drugs (NSAIDs) are inhibitors of COXs and act as analgesic, antipyretic and anti-inflammatory agents. It has been shown that some NSAIDs can exert an anti-tumor effect, and they have been used in preclinical trials [23,24,25,26,27,28]. Furthermore, from many case-control studies, it has emerged that the use of NSAIDs for a long time can reduce significantly the incidence of colorectal cancer and breast cancer [29,30]. In particular, celecoxib, a selective COX-2 inhibitor, characterized by a lower gastrointestinal toxic effect, is the only FDA-approved NSAID for patients with familial adenomatous polyposis (FAP) [31]. Moreover, in vitro and in vivo studies have shown that celecoxib exerts a chemopreventive and anticancer effect on several tumors, including melanoma [32,33,34].

Hence, in this study, we have evaluated the cytotoxic effect of celecoxib in two melanoma cell lines characterized by different histological origins. In particular, the action of celecoxib was evaluated in A2058 and SAN melanoma cell lines that derive from a lymph nodal metastasis [35] and primary tumor [36,37], respectively. We have found that celecoxib reduces cell viability and migratory capability and induces apoptosis of melanoma cells grown as two-dimensional (2D) cell cultures. Furthermore, celecoxib also exerts a toxic effect on three-dimensional (3D) cell cultures of melanoma cells.

## 2. Materials and Methods

### 2.1. Cell Cultures

The A2058 human melanoma cell line was provided by CEINGE (Naples, Italy) and the SAN human melanoma cell line was kindly gifted by Prof. Maria Fiammetta Romano of the University of Naples Federico II. Human primary fibroblasts were obtained and cultured as previously described [38] and their use received approval from the Ethics Committee of the University of Naples Federico II (Comitato Etico Università Federico II, Naples, Italy). The assigned protocol number is 228/18. A2058 and SAN melanoma cells were grown in Dulbecco’s Modified Eagle Medium (DMEM; Microgem Laboratory Research, Milan, Italy) supplemented with 10% Fetal Bovine Serum (FBS; Microgem Laboratory Research, Milan, Italy), 2 mM l-glutamine, 100 IU/mL penicillin G, and 100 µg/mL streptomycin in a humidified incubator at 37 °C under a 5% CO_2_ atmosphere. Cancer cells were split and seeded in plates (75 cm^2^) every 2 days and used for assays during the exponential phase of growth. Cell treatments were always carried out 24 h after plating. Cells were observed with a phase-contrast microscope, the Carl Zeiss (Jena, Germany) HBO 50/ac model; the images were acquired with a digital video camera (Panasonic Lumix DC-FZ82 Bridge) connected to the microscope.

### 2.2. Cell Viability Assay

Cell viability was measured using a CellTiter-Glo^®^ Luminescent Cell Viability Assay kit (Promega, Madison, WI, USA), as previously described [39]. Briefly, cells were seeded into 96-well microplates (1 × 10^4^ cells/well), and after 24 h of incubation, cells were treated with different concentrations of celecoxib or with 0.5% (*v*/*v*) DMSO (Sigma-Aldrich, St. Louis, MO, USA) as control vehicle. After 24, 48 or 72 h of treatment, the microplate was equilibrated at room temperature for 30 min prior to the addition of 100 μL/well of CellTiter-Glo. The plate was shaken for 2 min to induce cell lysis and incubated at room temperature for 10 min to stabilize the luminescent signal before reading. Luminescence was measured using a BioTek Synergy H1 microplate reader (Agilent, Santa Clara, CA, USA).

### 2.3. Colony Formation Assay

The colony-forming assay was performed as previously described [40]. Briefly, cells were seeded in duplicate in 6-well plates at a density of 4 × 10^2^ cells per well. After 2/3 days, cells were treated with 0.5% (*v*/*v*) DMSO as control vehicle or different celecoxib concentrations and incubated for an additional 7 days at 37 °C. Then, colonies were stained with 1% (*w*/*v*) crystal violet (Sigma-Aldrich, St. Louis, MO, USA) in 50% (*v*/*v*) ethanol for 1 h at room temperature. Cells were photographed with a digital camera (Panasonic Lumix DC-FZ82 Bridge); the number of colonies was counted using ImageJ 1.53t software.

### 2.4. Measurement of Caspase-3 Activity

The caspase-3 enzymatic activity was measured by using a caspase-3 fluorimetric assay kit (BioVision, Milpitas, CA, USA), according to the manufacturer’s protocol. Briefly, cells were seeded into 75 cm^2^ plates (3 × 10^6^ cells/plate) and after 24 h were treated for 7 h with different concentrations of celecoxib or 0.5% (*v*/*v*) DMSO as control vehicle. At the end of drug incubation, cells were collected, washed with phosphate-buffered saline (PBS, Sigma-Aldrich, St. Louis, MO, USA) constituted by 10 mM Na_2_HPO_4_, 2 mM KH_2_PO_4_, pH 7.4, supplemented with 137 mM NaCl and 2.7 mM KCl, and finally lysed at 4 °C in cell lysis buffer. Cell lysates were incubated with 50 µM DEVD-AFC substrates at 37 °C for 2 h, to detect caspase-3 activity, using a Cary Eclipse spectrofluorometer. Excitation and emission wavelengths were set at 400 nm and 505 nm, respectively; both excitation and emission slits were set at 10 nm.

### 2.5. Wound Healing Assay

The cell migration was evaluated by a wound assay. Briefly, cells (4 × 10^5^ cells/well) were seeded into 6-well plates and after 24 h of incubation the cell monolayer was carefully scratched with a sterilized pipette tip (p10) and incubated for 24 h with different concentrations of celecoxib or 0.5% (*v*/*v*) DMSO as control vehicle. To achieve an objective wound closure evaluation, eight fields per scratch were photographed and measured at 0 and 24 h with a digital video camera (Panasonic Lumix DC-FZ82 Bridge). Quantitative analysis of wound closure was performed by measuring the gap area by using ImageJ 1.53t software.

### 2.6. Generation and Growth of Spheroids

Melanoma cell spheroids were generated by using the previously described hanging-drops method [38]. Briefly, 20 μL of cell-suspension drop, supplemented with 0.25% methylcellulose (vol/vol in medium) and containing A2058 (5 × 10^4^ cells) or SAN (7 × 10^4^) cells were seeded on the inverted lids of 6-well plates containing 1 mL of PBS per well to avoid culture medium evaporation. After 48 h of incubation at 37 °C, 5μL of fresh culture medium was added to each drop to allow the growth of spheroids for a further 24 h at 37 °C.

### 2.7. Spheroid Migration Assay

Each single spheroid, generated as described above, was transferred from the lid of a 6-well plate to an individual well of a 24-well adhesion plate containing complete cell culture medium to allow spheroid adhesion. Spheroids were incubated for 3 days with different concentrations of celecoxib or 0.5% (*v*/*v*) DMSO as control vehicle. The area covered by melanoma cells migrating out from the spheroids and spreading on a plastic surface can be used as an index of cell migration [41]. To this aim, images were taken at different time points with a digital camera (Panasonic Lumix DC-FZ82 Bridge) connected to a phase-contrast microscope, Carl Zeiss (Jena, Germany) HBO 50/ac model.

### 2.8. 3D Hydrogel Invasion Assay

Tumor cell spheroids in hydrogel can be used to study, in vitro, the cell invasion processes [42]. The 3D hydrogel invasion assay was used to analyze the behavior of melanoma cells migrating from the spheroids into a matrix. Each single spheroid, generated as described above, was transferred from the lid of a 6-well plate to an individual well of a 96-well plate containing 100 µL/well of a mix of VitroGel Hydrogel Matrix (The Well Bioscience, North Brunswick, NJ, USA) and complete culture medium (ratio 2:1 (*v*/*v*)), according to the manufacturing protocol. After matrix solidification, 50 μL/well of complete culture medium was added and spheroids were incubated for 3 days with different concentrations of celecoxib or 0.5% (*v*/*v*) DMSO as control vehicle. Images were taken at different time points with a digital camera (Panasonic Lumix DC-FZ82 Bridge) connected to a phase-contrast microscope, Carl Zeiss (Jena, Germany) HBO 50/ac model.

### 2.9. Statistical Analysis

Data are reported as the mean ± standard error (SE). The statistical significance of differences among groups was evaluated using ANOVA, with the Bonferroni correction as a post hoc test or the Student t-test where appropriate. The significance was accepted at the level of *p* < 0.05.

## 3. Results and Discussion

### 3.1. Effect of Celecoxib on Morphology of Melanoma Cells

**Figure 1:**
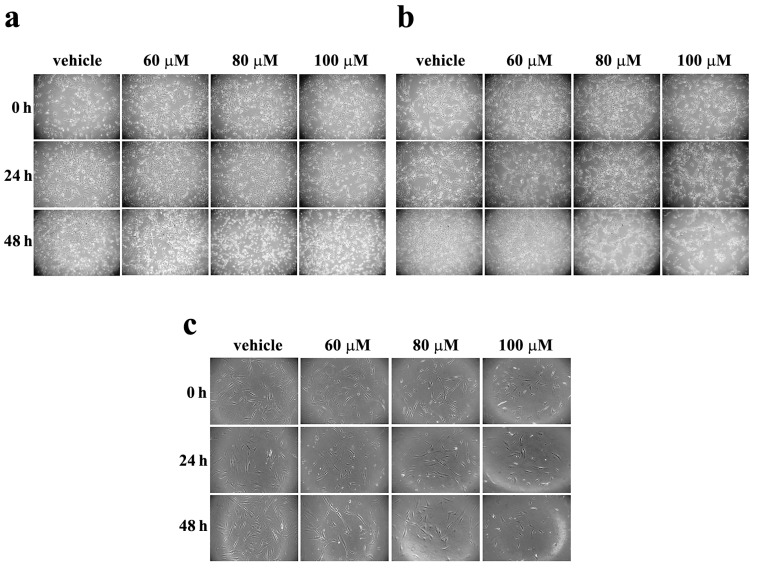
Effect of celecoxib on the cell morphology of A2058 and SAN melanoma cell lines and primary human fibroblasts. (**a**) A2058 melanoma cells, (**b**) SAN melanoma cells, and (**c**) primary human fibroblasts were treated with vehicle alone (0.5% (*v*/*v*) DMSO) or 60, 80 or 100 µM celecoxib for 24 and 48 h. Images are representative of three independent experiments. Magnification ×10.

A2058 and SAN melanoma cell lines were incubated with vehicle alone or with increasing concentrations of celecoxib (60, 80 or 100 μM) for 24 and 48 h, and morphological changes were monitored by a light microscopy (Figure 1).

The morphological changes were evident after 48-h treatment in both cell lines (Figure 1a,b). In particular, cell treatment with 80 and 100 µM celecoxib provoked cell morphological alterations that can be associated with a toxic action exerted by the drug. To investigate the specific toxic effect exerted by celecoxib in melanoma cells, human primary fibroblasts were incubated with the same concentrations of celecoxib for 24 and 48 h. As shown in Figure 1c, the morphology of fibroblasts does not appear to be greatly altered by treatment with celecoxib. The constant and ubiquitous presence of fibroblasts in any body tissue is well understood. Hence, the absence of significant morphological changes in fibroblasts treated with celecoxib suggests a more specific toxic effect of this drug on melanoma cells.

### 3.2. Effect of Celecoxib on Cell Viability of Melanoma Cells

**Figure 2:**
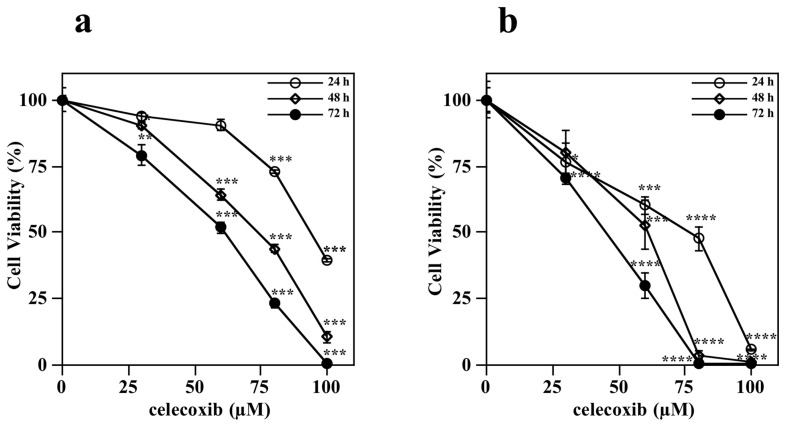
Dose- and time-dependent effect of celecoxib on melanoma cell viability. (**a**) A2058 cells and (**b**) SAN cells were incubated with vehicle alone (0.5% (*v*/*v*) DMSO) or 30, 60, 80 or 100 µM celecoxib. ATP assay was carried out and cell viability was evaluated after 24, 48 and 72 h of treatment. Values, reported as a percentage with respect to untreated control cells, represent the mean ± SE of three independent experiments performed in triplicate. * *p* < 0.05, ** *p* < 0.01, *** *p* < 0.001, **** *p* < 0.0001 compared to control cells.

The toxic effect exerted by celecoxib on melanoma cells was investigated by performing an ATP assay to evaluate the cell viability in the presence of the drug. In particular, A2058 and SAN melanoma cells were treated with vehicle alone or increasing concentrations of celecoxib (30, 60, 80 or 100 µM) for different incubation times. Data shown in Figure 2 indicate that celecoxib reduced the cell viability of both melanoma cell lines in a dose- and time-dependent manner.

In particular, in the A2058 cell line, after 72 h of incubation with celecoxib, the value of IC_50_ was 63 ± 4 µM and the maximum toxic effect was reached after 72-h incubation with 100 µM celecoxib (Figure 2a). On the other hand, SAN cells seem more sensitive to celecoxib compared to A2058 cells. Indeed, after 72 h of incubation with celecoxib the value of IC_50_ was 45 ± 4 µM and a complete absence of cell viability was already observed after 48-h treatment with 100 µM celecoxib (Figure 2b).

**Figure 3:**
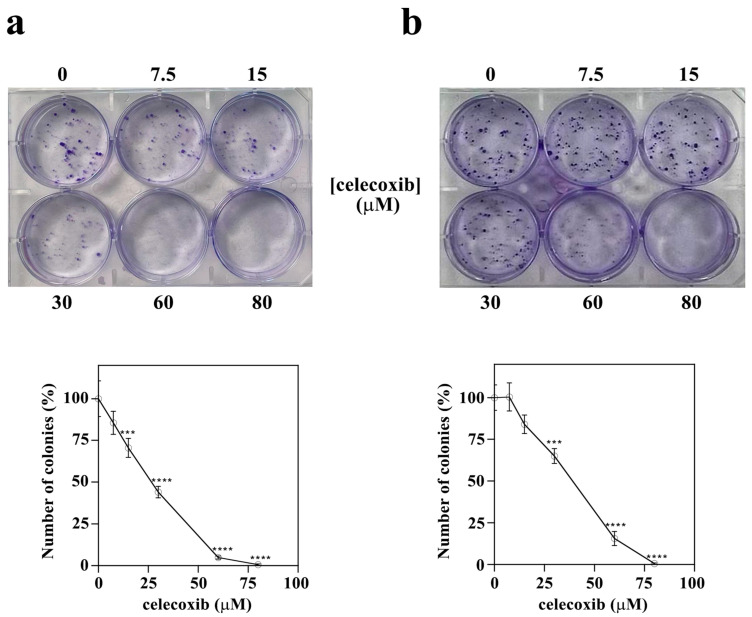
Effect of celecoxib on colony-forming ability of A2058 and SAN melanoma cells. (**a**) A2058 cells and (**b**) SAN cells were seeded into 6-well culture plates and treated with vehicle alone (0.5% (*v*/*v*) DMSO) or 7.5, 15, 30, 60 or 80 µM celecoxib for 7 days. Then, plates were photographed and images of representative experiments are shown. Plots report the number of colonies counted as indicated in the Materials and Methods Section; values are expressed in percentages with respect to untreated control cells and reported as the mean ± SE from at least three different experiments. *** *p* < 0.001, and **** *p* < 0.0001 compared to control cells.

To further evaluate celecoxib’s cytotoxic action, a colony-forming assay was performed. This test is widely used in cancer research, as the capacity to form clones represents a typical trait of cancer cells, and it is a standard tool to evaluate, for the long term, the cytotoxic effects of various agents with potential clinical application [43]. Hence, A2058 and SAN melanoma cells were treated with vehicle alone or increasing concentrations of celecoxib (7.5, 15, 30, 60 or 80 µM) and the number of colonies was detected (Figure 3).

In particular, celecoxib affected the ability to form colonies in both cell lines, even at low concentrations. Indeed, in both A2058 (Figure 3a) and SAN cells (Figure 3b) a significant reduction in the number of colonies was already observed in the presence of 15 or 30 µM celecoxib, respectively, and in both cell lines 80 µM celecoxib completely inhibited the formation of colonies. Furthermore, celecoxib also exerts an effect on the size of colonies. In particular, colonies of A2058 cells (Figure 3a) and, to a lesser extent, of SAN cells (Figure 3b), were smaller with respect to control groups. It is important to note that, in vitro, colonies can show different morphology, and for this reason they can be classified as holoclones, meroclones, and paraclones [44]. These colony types can derive from stem cells, transit-amplifying cells, and differentiated cells, respectively. In particular, by using prostate cancer cells, it has been shown that the proportion of stem cells, rather than their presence or absence, is diverse in the different colony types [45]. Hence, it can be supposed that the smaller size of colonies induced by celecoxib could be indicative of an effect of the drug on cancer stem cells. Further experiments will be performed to evaluate this hypothesis.

**Figure 4:**
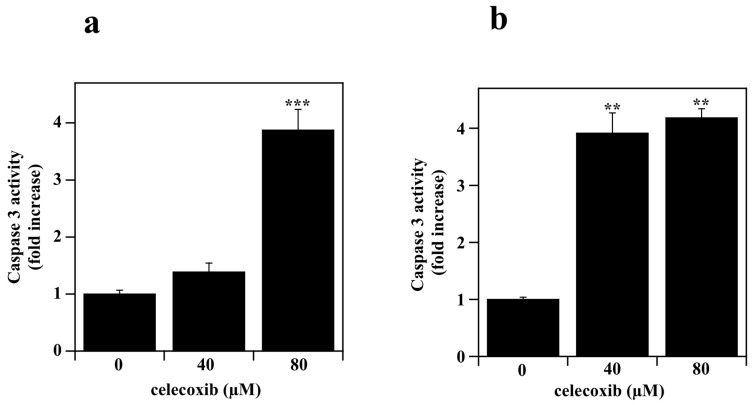
Effect of celecoxib on the caspase-3 activity in melanoma cell lines. (**a**) A2058 cells and (**b**) SAN cells were incubated with vehicle alone (0.5% (*v*/*v*) DMSO) or 40 or 80 µM celecoxib. Caspase-3 enzymatic activity is expressed as a fold increase with respect to untreated control cells and reported as the mean ± SE from at least three different experiments. ** *p* < 0.01, *** *p* < 0.001 compared to control cells.

It is known that many drugs, including NSAIDs, exert their cytotoxic effects through the induction of apoptosis [24,46,47]. Hence, the pro-apoptotic action of celecoxib was investigated by measuring the caspase-3 enzymatic activity in melanoma cells. The effect of celecoxib on the activation of caspase-3, the main final mediator of the apoptosis program [48], was monitored after drug incubation. In particular, A2058 and SAN melanoma cells were treated with vehicle alone or different concentrations of celecoxib (40 or 80 µM) for 7 h and caspase-3 enzymatic activity was measured fluorometrically (Figure 4).

A significant increase of caspase-3 activity was induced in both cell lines by treatment with 80 µM celecoxib. On the other hand, the effect of celecoxib at 40 µM was significant only in SAN cells (Figure 4b), although the trend towards the increase was also evident in A2058 cells (Figure 4a).

Taken together, these data demonstrate that celecoxib exerts a strong toxic effect on A2058 and SAN melanoma cell lines and that this drug can affect melanoma cell viability by inducing apoptosis.

### 3.3. Effect of Celecoxib on Motility Capacity of Melanoma Cells

**Figure 5:**
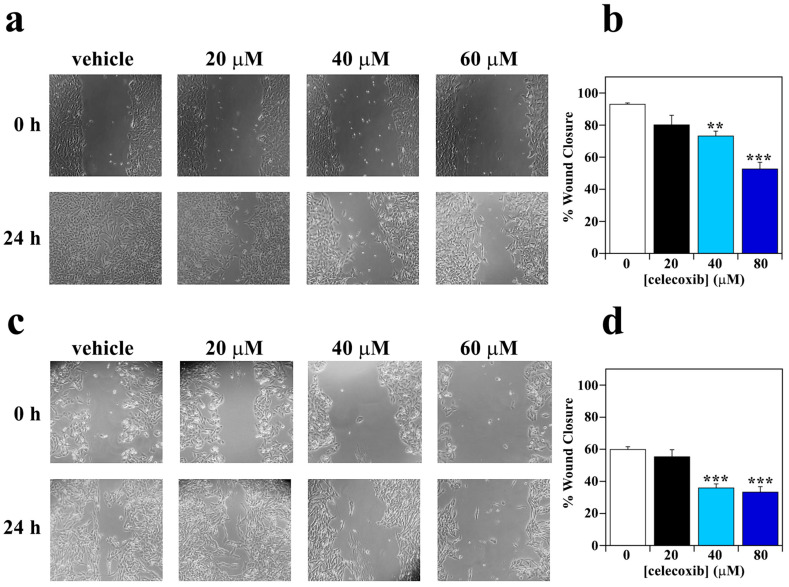
Effect of celecoxib on migratory capability of melanoma cells. (**a**) A2058 cells and (**c**) SAN cells were wounded with a pipette tip and photographed immediately after the wounding (0 h) and after 24 h of treatment with vehicle alone (0.5% (*v*/*v*) DMSO) or 20, 40 or 60 µM celecoxib. Quantification of migration capacity of (**b**) A2058 cells and (**d**) SAN cells incubated for 24 h with vehicle alone (0.5% (*v*/*v*) DMSO) (white bar), 20 µM celecoxib (black bar), 40 µM celecoxib (cyan bar), or 60 µM celecoxib (blue bar). Eight fields per scratch were measured to achieve an objective evaluation. Data are expressed as percentage of wound closure after 24 h of treatment with respect to 0 h and reported as the mean of two independent experiments ± SE. ** *p* < 0.01; *** *p* < 0.0001 compared to control cells.

Melanoma cells are characterized by a high migratory and invasive capacity that represents a requisite for metastasis [49,50]. It has been shown that prostaglandins regulate the migration and invasion of tumor cells [51]. Hence, we have evaluated the action of celecoxib on melanoma cell motility through a wound-healing assay. In particular, the motility of A2058 and SAN melanoma cells was monitored after 24 h of treatment with different concentrations of celecoxib. As shown in Figure 5, the effect of celecoxib on the migratory capability of melanoma cells was dose-dependent, with a similar trend in A2058 (Figure 5a,b) and SAN (Figure 5c,d) melanoma cells.

In particular, a not significant decrease of wound closure percentage was already evident in the presence of 20 µM celecoxib. This decrease became significant after treatment with 40 or 60 µM celecoxib. Therefore, it is noteworthy that celecoxib also exerts an anti-migratory action on melanoma cells. It is important to note that in 27 patients, affected by non-resectable metastatic melanoma and treated with celecoxib, the median overall survival was increased by up to 31.9 months. Moreover, among these patients, one complete response was also observed [52].

### 3.4. Effect of Celecoxib on 3D Melanoma Cell Cultures

**Figure 6:**
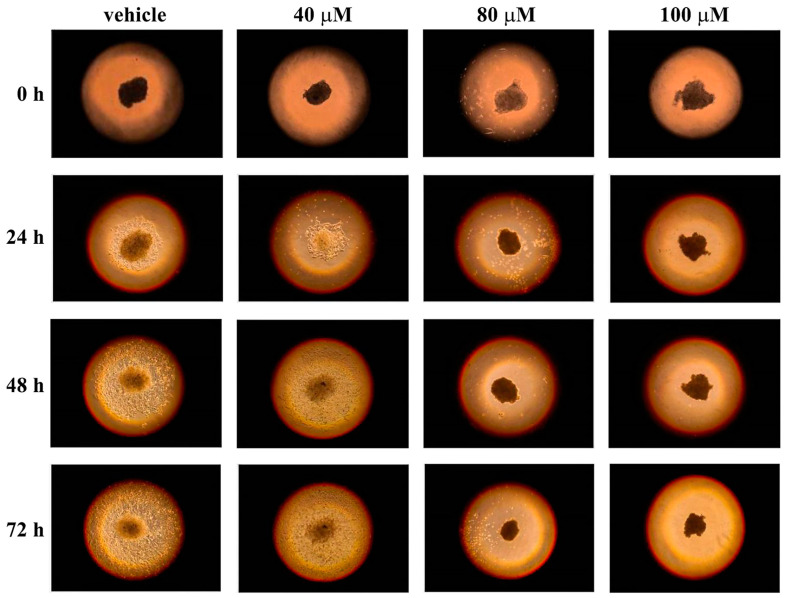
Influence of celecoxib on cell outgrowth and migration from spheroids of A2058 cells. Single spheroids, placed into individual wells of a 24-well adhesion plate, were treated with vehicle alone (0.5% (*v*/*v*) DMSO) or with increasing concentrations of celecoxib (40, 80 or 100 µM). The cell outgrowth from each spheroid was monitored after 24, 48 and 72 h of treatment. Images are representative of three independent experiments. Magnification ×10.

**Figure 7:**
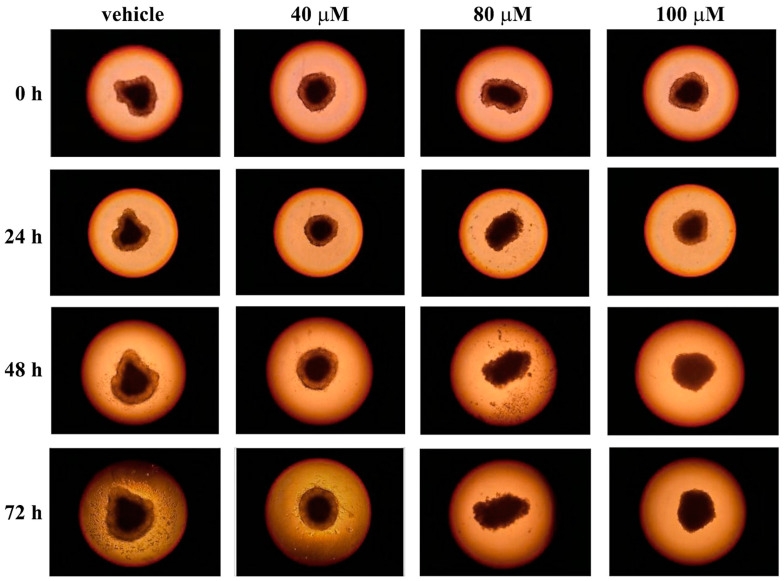
Influence of celecoxib on cell outgrowth and migration from spheroids of SAN cells. Single spheroids, placed into individual wells of a 24-well adhesion plate, were treated with vehicle alone (0.5% (*v*/*v*) DMSO) or with increasing concentrations of celecoxib (40, 80 or 100 µM). The cell outgrowth from spheroid was monitored after 24, 48 and 72 h of treatment. Images are representative of three independent experiments. Magnification ×10.

The use of two-dimensional (2D) cell cultures represents a valid tool to evaluate the cytotoxicity of molecules, but it also represents a preliminary step to test the cell’s sensibility to drugs. Indeed, cells in a 2D model grow on a flat surface, relying both on cell–cell interaction and cell–plate adhesion, which can affect many cellular processes [53]. As a result, cells cultured in 2D may not behave as they would in the body, because this model does not adequately mimic the in vivo microenvironment. Hence, three-dimensional (3D) cell cultures, such as spheroids, may be an effective system to use as a drug testing platform, being a compromise between 2D cell cultures and animal models. In particular, spheroids are an important experimental system for cancer research because they show similar features to tumor tissue organization [54]. Furthermore, many studies have reported different effects on 2D cell cultures and spheroids in terms of drug resistance, showing that spheroids, as the in vivo models, are often more drug-resistant than 2D cell cultures [55]. Hence, to better investigate the anticancer action of celecoxib, this drug was tested on 3D cell cultures of A2058 and SAN melanoma cells. To this end, we generated spheroids by using the hanging-drops method and, after 72 h, each drop containing one spheroid was transferred into a standard adhesion well of a 24-well plate. Spheroids were left untreated and treated with different concentrations of celecoxib (40, 80 or 100 µM), and the cell adhesion and outgrowth from spheroids was monitored after 24, 48 and 72 h. As shown in Figure 6 and Figure 7, celecoxib exerted a strong toxic effect at 80 and 100 µM in both cell lines.

Indeed, after treatment with 80 and 100 µM of celecoxib, the cell outgrowth from the spheroids is clearly absent. In the presence of 40 µM of celecoxib, the behavior of the two cell types is different. Indeed, A2058 melanoma cells seem more resistant to 40 µM of celecoxib, compared with SAN cells (Figure 6 and Figure 7), because the area of cell outgrowth from the spheroids after 48 and 72 h was only slightly less expanded with respect to the control. On the other hand, after 48 h and to a greater extent after 72 h of incubation, the outgrowth of SAN cells from the spheroids was lower with respect to the control (Figure 7). These results indicate that the reduction or the absence of cell outgrowth from the spheroids induced by celecoxib could be associated with an inhibitory effect of the drug on cell migratory capability.

**Figure 8:**
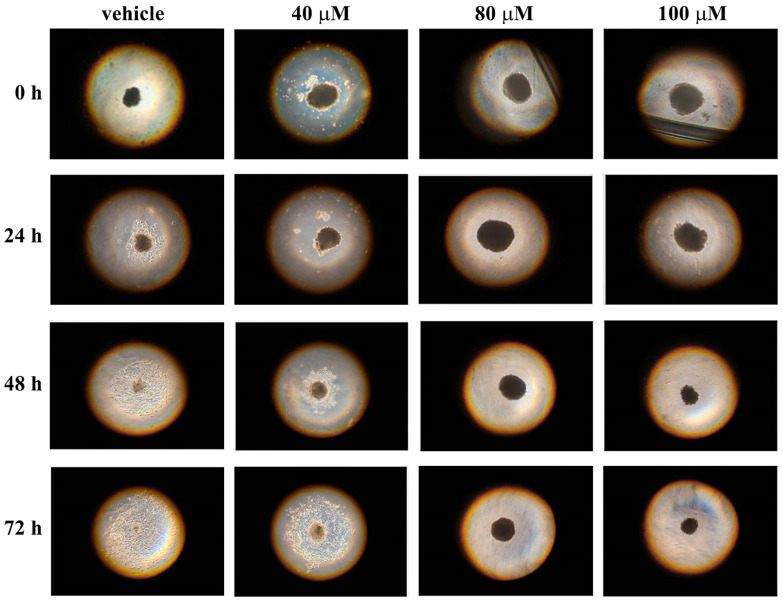
Influence of celecoxib on A2058 cell invasion capacity from spheroid into hydrogel. Single spheroids of A2058 cells, transferred into an individual well of a 96-well plate containing hydrogel, were treated with vehicle alone (0.5% (*v*/*v*) DMSO) or with increasing concentrations of celecoxib (40, 80 or 100 µM), and the cell invasion capacity from spheroid in the surrounding hydrogel was monitored after 24, 48 and 72 h of treatment. Images are representative of three independent experiments. Magnification ×10.

**Figure 9:**
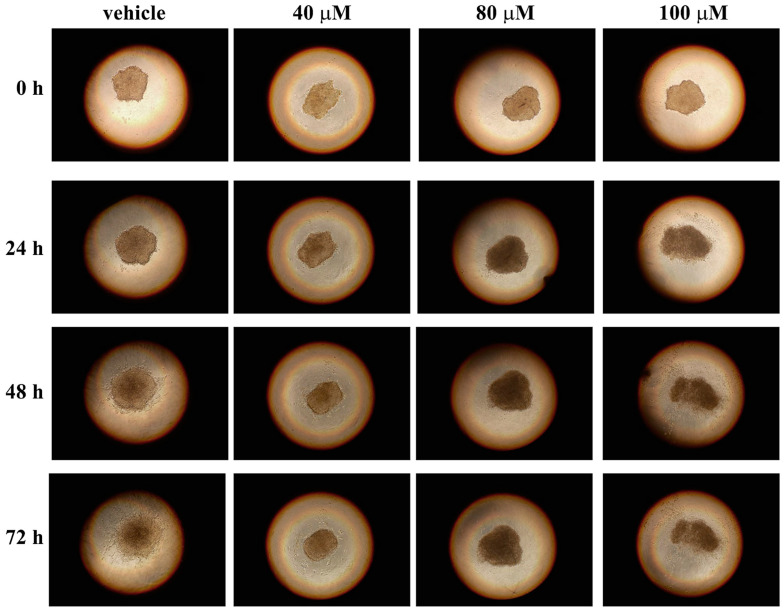
Influence of celecoxib on SAN cell invasion capacity from spheroid into hydrogel. Single spheroids of SAN cells, transferred into an individual well of a 96-well plate containing hydrogel, were treated with vehicle alone (0.5% (*v*/*v*) DMSO) or with increasing concentrations of celecoxib (40, 80 or 100 µM) and the cell invasion capacity from spheroid in the surrounding hydrogel was monitored after 24, 48 and 72 h of treatment. Images are representative of three independent experiments. Magnification ×10.

We have also used 3D cell cultures of melanoma cells to evaluate the action of celecoxib on cell invasion capacity. To this aim, single spheroids, produced by using the hanging-drops method, were transferred after 72 h into individual wells of a 96-well plate containing hydrogel. Hydrogels represent a good tool to study the growth, as well as the response, of tumor spheroids to drugs in a more appropriate environment [40,56,57]. In particular, the hydrogel polymerization forms a dense gel that encapsulates the spheroid, permitting the study of cell invasion behavior [58,59,60]. Hence, spheroids of A2058 and SAN melanoma cells were untreated and treated with different concentrations of celecoxib (40, 80 or 100 µM) and their cell invasion capability into hydrogel was evaluated after 24, 48 and 72 h (Figure 8 and Figure 9).

In particular, the results evidenced a gradual increase of cell invasion from the spheroids of A2058 cells into the hydrogel in untreated samples (Figure 8). Indeed, the area of invasion was already evident after 24 h and increased after 48 and 72 h. On the other hand, in samples treated with 40 µM celecoxib, the cell invasion began to be clear only after 48 h and the invasion area, also after 72 h of treatment, was less pronounced with respect to the control. Furthermore, in the presence of 80 and 100 µM celecoxib, no cell invasion from spheroid into hydrogel was evident. In untreated SAN cells, the process of invasion was evident only after 48 h and increased at 72 h (Figure 9). In the presence of 40 µM celecoxib, the area of cell invasion was little pronounced after 48 and 72 h of drug treatment, and also in this melanoma cell line, treatment with 80 and 100 µM of celecoxib inhibited completely the cell invasion into the hydrogel. These data indicate that celecoxib also exerts an inhibitory effect on both A2058 and SAN cell invasion capability, although SAN cells also, in this test, are more sensible to celecoxib with respect to A2058 cells.

Taken together, these experiments indicate that celecoxib is capable of exerting a toxic action also when it is tested on a 3D cell structure such as a spheroid of melanoma cells. Hence, a possible use of celecoxib in clinical trials, alone or in association with other anti-melanoma drugs, could be considered.

## 4. Conclusions

The results obtained in this study highlight the toxic effect exerted by celecoxib in human melanoma cell lines. In particular, the cytotoxic action of celecoxib was evidenced in both 2D and 3D cultures of melanoma cells. Generally, the anti-tumor effect of some molecules was first evaluated by using 2D cell cultures. However, the results obtained by using such models can be clinically unreliable or ineffective and must be validated in a system that more resembles in vivo conditions [55]. Hence, a better validation of the toxic effects of drugs can be obtained by using 3D cultures. Therefore, the additional results obtained when celecoxib was tested also on melanoma spheroids can be very informative for the study of celecoxib in animal models and/or clinical trials. However, further investigations will be performed to deeper evaluate the action of this drug on melanoma cell growth, also at the molecular level. Finally, it will be interesting to study the effects of celecoxib in association with other conventional drugs used in melanoma therapy.

## Data Availability

Not applicable.
